# Imaging Cell Competition in Ex-Vivo *Drosophila* Adult Brains

**DOI:** 10.3390/mps9010001

**Published:** 2025-12-20

**Authors:** Andrés Gutiérrez-García, Mariana Marques-Reis, Eduardo Moreno

**Affiliations:** 1 Cell Fitness Lab, Champalimaud Centre for the Unknown, Av. Brasília, 1400-038 Lisbon, Portugal; 2 Department of Cell & Developmental Biology, Feinberg School of Medicine, Northwestern University, Chicago, IL 60611, USA

**Keywords:** cell competition, *Drosophila melanogaster*, live imaging

## Abstract

Live imaging has been instrumental in understanding cellular dynamics in *Drosophila* tissues, but technical limitations have prevented the long-term visualization of cell competition in adult brains. Here, we describe a simple ex vivo protocol that enables extended live imaging of adult *Drosophila* brains for up to 32 h. The method relies on non-supplemented Schneider’s *Drosophila* medium and hydrophobic interactions to maintain brain stability during imaging, eliminating the need for complex culture conditions or embedding procedures. We validate this approach by studying cell competition in the optic lobes following traumatic brain injury, where cell competition is expected to occur with a peak at 48 h after damage. We demonstrate the value of this method by visualizing the expression of the fitness checkpoint Azot in a loser cell and its subsequent elimination. This protocol offers a versatile platform for studying cell competition and other cellular processes requiring extended observation of the adult *Drosophila* brain.

## 1. Introduction

The Darwinian principle of ‘survival of the fittest’ extends beyond macroscopic organisms to the cellular level through a phenomenon known as Cell Competition. This process, first described in 1975 in *Drosophila* wing imaginal disks, demonstrated that wild-type cells could outcompete slow-growing cells carrying mutations in *minute* genes [1]. Since then, the concept has evolved to encompass the elimination of viable but suboptimal cells when they encounter cells with superior fitness within the same tissue compartment. This fundamental biological process is conserved from *Drosophila* to mammals, including humans [2,3]. Cell competition can be triggered by various factors, including competition for survival factors [4,5], mechanical forces [6,7], and differential expression of fitness markers [8,9,10,11].

Among these fitness markers, the Flower protein family plays a crucial role in cellular fitness communication. Conserved across *Drosophila*, mice, and humans, the Flower protein in *Drosophila* exists in three isoforms: Flower ubi, present in cells with higher fitness status (‘winners’), and Flower Lose A and Flower Lose B, present in cells with lower fitness status (‘losers’) [8,12,13]. Notably, cell elimination occurs only in heterogeneous populations where cells of different fitness levels interact [8,14]. In such contexts, loser cells activate the fitness checkpoint protein Azot, which triggers the pro-apoptotic gene *hid*, ultimately leading to programmed cell death [15].

While previous studies have demonstrated the co-expression of Flower Lose B and Azot in fixed tissue samples, particularly in the adult brain, the temporal dynamics of this process remain poorly understood [16]. Specifically, the time course from initial Flower Lose B expression to Azot activation and subsequent cell elimination requires further investigation. Ex vivo live imaging studies of cell competition in *Drosophila* have been primarily confined to larval imaginal disks [17,18], while other ex vivo imaging approaches have been established for larval and pupal brains in different contexts, typically for periods less than 18 h [19,20]. However, competitive events require longer observation windows, as the comparison of fitness status, the decision-making process for cell elimination, and the execution of cell death are multi-step processes. Current in vivo techniques using imaging windows allow visualization of the midgut and certain regions of the adult brain, but these methods are optimized for light-sheet microscopy or two-photon microscopy rather than confocal imaging [21,22,23,24]. For adult brains, confocal microscopy can offer superior spatial resolution and more straightforward multicolor imaging workflows than traditional light-sheet microscopy, while avoiding the complexity of two-photon systems [25]. Recent efforts have been made to study ex vivo *Drosophila* adult brains with confocal microscopy, but they still require complex pumping systems that might be unnecessary if no pharmacological manipulation is needed [26].

To address these limitations, we have developed a simplified ex vivo imaging approach that enables the long-term observation (32 h) of adult *Drosophila* brains. Our system utilizes a non-supplemented Schneider’s *Drosophila* Medium and leverages hydrophobic interactions between the brain tissue and the imaging chamber to maintain specimen stability. To induce competitive events, we performed traumatic brain injury (TBI) in the right optic lobe, as this injury triggers an initial wave of apoptosis due to mechanical damage, followed by a second wave of cell elimination peaking at 48 h post-injury that is driven by flower-dependent cell competition [27,28]. This methodology will not only facilitate the study of cell competition dynamics but also provide a versatile platform for extended ex vivo imaging of the adult *Drosophila* brain.

## 2. Experimental Design

**Figure 1:**
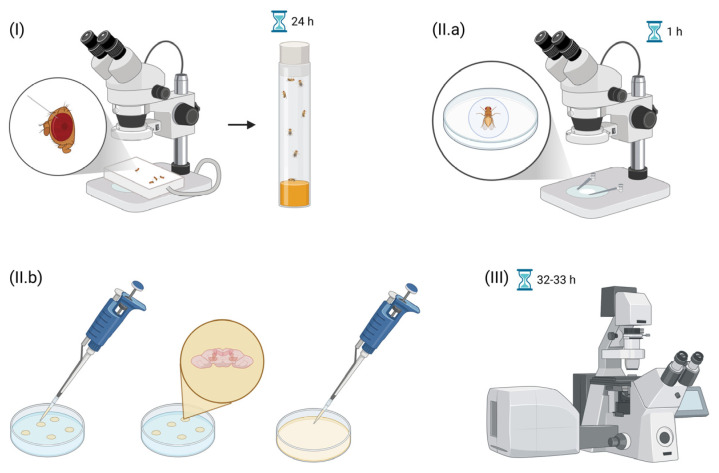
Schematic representation of the live imaging in ex vivo Drosophila adult brains. (**I**) Drosophila husbandry and preparation for experiments, specifically the TBI and the 24 h period for fly recovery. (**II.a**) Drosophila adult brain dissection. (**II.b**) Attachment of the brain to the bottom of the Nunc™ Glass Bottom Dish. (**III**) Imaging session in the confocal microscope. Created in BioRender. Gutierrez, A. (2025) https://BioRender.com/qfytp6d.

In this protocol, we describe the experimental setup and procedures for live imaging of ex vivo adult *Drosophila melanogaster* brains following TBI, enabling the observation of cell competition events through fitness fingerprint comparisons (Figure 1). Although developed for studies of cell competition and tissue remodeling after TBI, the sample preparation and imaging procedures described here can be broadly applied to any ex vivo live imaging study of adult *Drosophila* brains.

### 2.1. Reagents

1. Schneider’s *Drosophila* Medium (Biowest, Nuaillé, France; Cat. No.: L0207-500).

### 2.2. Materials

2. PIPETMAN P1000, 100–1000 µL, Metal Ejector (Gilson, Middleton, WI, USA; Cat. No.: F144059M).
3. PIPETMAN P200, 20–200 µL, Metal Ejector (Gilson, Middleton, WI, USA; Cat. No.: F144058M).
4. Pipette Tips SureOne™ Micropoint, Universal Fit, Non-Filtered 200 µL, 1000 µL (Thermo Scientific, Waltham, MA, USA; Cat. No.: 02-707-410; 02-707-407).
5. Minutien Pins (Fine Science Tools, Heidelberg, Germany, Cat. No.: 26002-10).
6. Petrolatum (Unilever, NJ, USA; Cat. No.: 305212352009).
7. Sterilin™ 30 to 140 mm Petri Dishes (Thermo Scientific, Waltham, MA, USA; Cat. No.: PF55).
8. Fine Forceps (Fine Science Tools, Heidelberg, Germany, Cat. No.: 11403-11).
9. Nunc™ Glass Bottom Dishes (Thermo Scientific, Waltham, MA, USA; Cat. No.: 150682).

### 2.3. Drosophila Melanogaster Lines

10. *flower{KO; KI-flowerLoseB::mCherry}* [8].
11. *azot{KO; KI-LexA::p65}* [16].
12. *26xLexAop-CD8::GFP* (Bloomington Drosophila Stock Center, Bloomington, IN, USA; Cat. No.: 32207).

### 2.4. Equipment

- Flystuff^®^ Drosophila Anesthesia Systems (Genesee Scientific, El Cajon, CA, USA; Cat. No.: 59-121C).
- Zeiss Stemi 305 Stereo zoom microscope (ZEISS Microscopy, Oberkochen, Germany, Cat. No.: 435063-9020-100).
- Zeiss LSM 880 Airyscan Inverted Confocal Microscope with stage incubator (ZEISS Microscopy, Oberkochen, Germany).
- Objective Plan-Apochromat 40x/1.4 Oil DIC M27 (ZEISS Microscopy, Oberkochen, Germany, Cat. No.: 420762-9900-000).

## 3. Procedure

### 3.1. Drosophila Husbandry and Preparation for Experiments (Duration: Minimum of 13 Days)

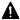
 **CRITICAL STEP:** Start by preparing the fly stocks needed for the experiment. In our experimental conditions, we first developed the stocks *w* ; *azot*{*KO; KI-LexA::p65*}/*CyO*, *26xLexAop-CD8::GFP/Tm6B* and *w*; ; *flower{KO; KI-flowerLoseB::mCherry*}/*Tm6B* from the lines mentioned in Section 2.3.

1. Cross 3 males of the genotype *w*; *azot{KO; KI-LexA::p65}/CyO*, *26xLexAop-CD8::GFP/Tm6B* with 10 female virgins of the genotype *w*; ; *flower{KO; KI-flowerLoseB::mCherry}/Tm6B*. Keep the cross in wide plastic vials with Vienna standard media [29] in a chamber with a controlled temperature of 25 °C and humidity of 70%.
2. Discard the adults after 6 days.
  **OPTIONAL STEP:** These adults can be put in a new vial to establish a new cross in case it is needed for another experiment.
3. The F1 generation should hatch 10 days after the cross. Collect the recently hatched flies into a new vial.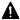 **CRITICAL STEP:** Wait 2 days to perform the TBI on these flies.
4. Use a minutien pin to stab the young adult flies (2 days old) in the right optic lobe, through the cuticle on the dorsal part of the head (as described in [27]), next to the second pair of bristles that surround the retina.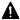 **CRITICAL STEP:** Wait 24 h before starting the dissections.

### 3.2. Drosophila Adult Brain Dissection (Duration: 1 h)

We followed the principles stated in [30] with small modifications.

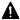
 **CRITICAL STEP:** Before the dissections, place the Schneider’s *Drosophila* Medium in a 4 °C chamber.

1. Prepare 15 mL of chilled Schneider’s *Drosophila* Medium and place it on ice.
2. Coat a Petri dish with a thin layer of *Petrolatum*.
3. Anesthetize the flies with CO_2_ and, with the forceps, place them belly up in the Petri dish with the wings embedded in *Petrolatum.*
4. Fill the Petri dish with chilled Schneider’s *Drosophila* Medium.
5. With the forceps, detach the proboscis and grab from below the cavity with each forcep, break the head cuticle, and expose the brain.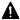 **CRITICAL STEP:** At this point, the brain is still attached to the rest of the body.
6. Clean the brain from the surrounding trachea without damaging its structure.
7. Repeat steps 1–6 until the desired number of brains is obtained (in our case, we dissected 5 brains).
8. In a Nunc™ Glass Bottom Dish, place 40 μL droplets of chilled Schneider’s *Drosophila* Medium.
9. Use the forceps to detach the brain from the body, perform final cleaning if necessary, and transfer each brain to a droplet in the Nunc™ Glass Bottom Dish.
10. Place each brain at the bottom of each droplet, facing down.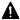 **CRITICAL STEP:** The brain–glass adhesion relies upon hydrophobic contact with the glass. The hydrophobic contact is very fragile, so it is advisable to dissect more than one brain.
11. Gently fill the Nunc™ Glass Bottom Dish with 5 mL of chilled Schneider’s *Drosophila* Medium.

**Troubleshooting:**

1. The droplets provide mechanical stability to the brain, reducing susceptibility to turbulence and preventing detachment. We observed that embedding the brains in droplets before filling the Nunc™ Glass Bottom Dish with 5 mL of chilled Schneider’s Drosophila Medium visibly decreased the likelihood of tissue detachment compared to adding the brains after the dish had been filled.

### 3.3. Imaging (Duration: 32 to 33 h)

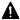
 **CRITICAL STEP:** Set the stage-top incubator to 25 °C

1. Follow the instructions for imaging according to your laboratory’s system. In our case, we used the Zeiss LSM 880 Airyscan confocal microscope, and the images were taken with the objective Plan-Apochromat 40x/1.4 Oil DIC M27 and a frame size of 2048 × 2048. The “GFP” and “Flower LoseB” channels refer to the specific ranges of wavelengths chosen for redirection to the detector. For the “GFP” channel, we selected the range from 499 nm to 548 nm, and for the “Flower LoseB” channel, we selected the range from 565 nm to 644 nm. The depth of the Z-stack was 40 μm, the step between frames was 1 μm, and each frame of the video corresponded to 15 min for a total duration of 32 h from the beginning of the imaging session.

**Troubleshooting:**

1. We imaged one brain per session to minimize the Nunc™ Glass Bottom Dish movement and prevent tissue detachment. To ensure the robustness of the protocol, we repeated the imaging session three independent times.

## 4. Expected Results and Discussion

**Figure 2:**
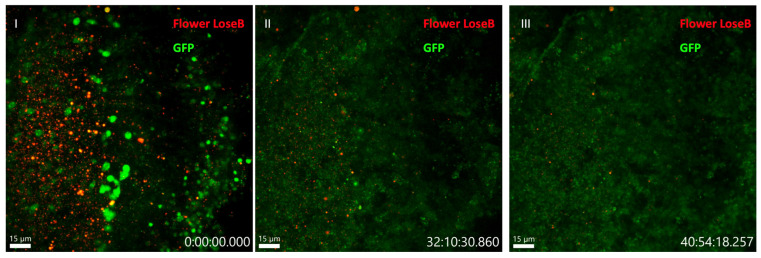
Viability of the Drosophila adult brain tissue up to 32 h after traumatic brain injury. First frame (**I**), 32 h (**II**) and last frame at 40 h (**III**) showing that there are still competitive events at 32 h, but not at 40 h since the beginning of the imaging session. Flower LoseB is represented in red, and GFP in green. 40-µm image projection. The red scale bar indicates 15 µm. Genotype: ywf; azot{KO; KI-LexA::p65}/+; 26xLexAop-CD8::GFP, flower{KO; KI-flowerLoseB::mCherry}/+.

**Figure 3:**
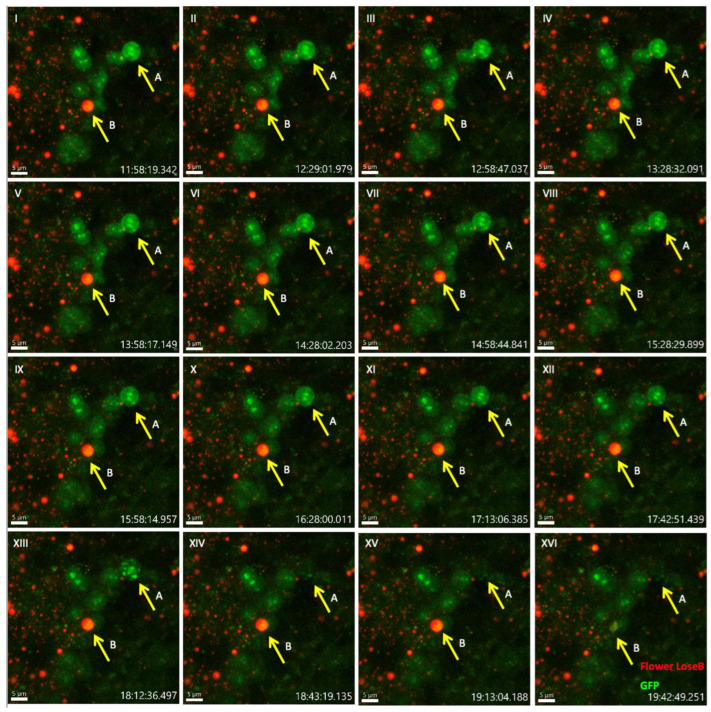
Live imaging of ex vivo Drosophila adult brain during Flower-dependent cell competition: (**I**–**XVI**) show insets of the damaged optic lobe from 36 to 40 h after damage (12 to 20 h after the beginning of the imaging session), in intervals of 30 min for simplicity, although the acquisition was every 15 min as described in the procedures. Flower LoseB is represented in red, and GFP in green. The yellow arrows indicate the loser cells of interest (A and B). 40-µm image projection. The red scale bar indicates 5 µm. Genotype: ywf; azot{KO; KI-LexA::p65}/+; 26xLexAop-CD8::GFP, flower{KO; KI-flowerLoseB::mCherry}/+.

The ex vivo system successfully maintained brain tissue viability for 32 h, as evidenced by the persistent fluorescent signal throughout the imaging period (Figure 2). From 32 to 40 h, we ceased to see competitive events. To analyze cell competition dynamics, we focused on a temporal window between 36 and 42 h post-injury (corresponding to 12 to 20 h since the beginning of the imaging session), when most competitive interactions are expected to occur [28] (Figure 3, Supplementary Video S1).

To visualize these competitive events, we employed a dual-labeling system: Flower LoseB was tagged with mCherry, while Azot expression was monitored using the bipartite LexA-LexAop system driving GFP expression [16]. Our time-lapse imaging revealed two cellular behaviors. In the initial frame (Figure 3I), we identified two cells of interest: cell A expressing only Azot (GFP-positive) and cell B co-expressing both Flower LoseB (mCherry-positive) and Azot (GFP-positive). Between frames (XIII) and (XIV), cell A exhibited a morphological disruption and disappearance, and between frames (XV) and (XVI), one can observe the morphological disruption and disappearance of cell B. These disappearances suggest apoptotic events, as Azot precedes *hid* activation and consequent cell apoptosis [15], aligned with the dynamics of Flower-dependent cell competition [15,16]. However, definitive confirmation of apoptosis would require additional cell death markers not included in our current setup.

This ex vivo imaging approach complements existing in vitro competition assays by enabling the direct visualization of competitive cell behaviors in intact brain tissue [13]. The straightforward nature of our protocol, requiring only standard laboratory equipment and commercially available *Drosophila* culture medium, makes it readily adaptable for any *Drosophila* research laboratory interested in studying cellular dynamics in the adult brain.

## Data Availability

Live imaging raw data is available upon request.

## Supplementary Materials

The following supporting information can be downloaded at: https://www.mdpi.com/article/10.3390/mps9010001/s1, Video S1: Video of Live Imaging of ex-vivo *Drosophila* adult brain during Flower-dependent cell competition after traumatic brain injury from 12 to 20 h after the beginning of the imaging session. Flower LoseB is represented in red, and GFP in green. The yellow arrows indicate the loser cells of interest (A and B). 40-µm image projection. The red scale bar indicates 5 µm. Genotype: *ywf*; *azot{KO; KI-LexA::p65}*/+; *26xLexAop-CD8::GFP*, *flower{KO; KI-flowerLoseB::mCherry}/+.*

## Abbreviations

The following abbreviations are used in this manuscript:

GFP	Green Fluorescent Protein
KI	Knock-in
KO	Knock-out
TBI	Traumatic Brain Injury

## References

[1] Morata G., Ripoll P. (1975). Minutes: Mutants of Drosophila Autonomously Affecting Cell Division Rate. Dev. Biol..

[2] Morata G. (2021). Cell Competition: A Historical Perspective. Dev. Biol..

[3] Cong B., Cagan R.L. (2024). Cell Competition and Cancer from Drosophila to Mammals. Oncogenesis.

[4] Moreno E., Basler K., Morata G. (2002). Cells Compete for Decapentaplegic Survival Factor to Prevent Apoptosis in Drosophila Wing Development. Nature.

[5] Martins V.C., Busch K., Juraeva D., Blum C., Ludwig C., Rasche V., Lasitschka F., Mastitsky S.E., Brors B., Hielscher T. (2014). Cell Competition Is a Tumour Suppressor Mechanism in the Thymus. Nature.

[6] Brás-Pereira C., Moreno E. (2018). Mechanical Cell Competition. Curr. Opin. Cell Biol..

[7] Wagstaff L., Goschorska M., Kozyrska K., Duclos G., Kucinski I., Chessel A., Hampton-O’Neil L., Bradshaw C.R., Allen G.E., Rawlins E.L. (2016). Mechanical Cell Competition Kills Cells via Induction of Lethal P53 Levels. Nat. Commun..

[8] Rhiner C., López-Gay J.M., Soldini D., Casas-Tinto S., Martín F.A., Lombardía L., Moreno E. (2010). Flower Forms an Extracellular Code That Reveals the Fitness of a Cell to Its Neighbors in Drosophila. Dev. Cell.

[9] Yamamoto M., Ohsawa S., Kunimasa K., Igaki T. (2017). The Ligand Sas and Its Receptor PTP10D Drive Tumour-Suppressive Cell Competition. Nature.

[10] Akieda Y., Ogamino S., Furuie H., Ishitani S., Akiyoshi R., Nogami J., Masuda T., Shimizu N., Ohkawa Y., Ishitani T. (2019). Cell Competition Corrects Noisy Wnt Morphogen Gradients to Achieve Robust Patterning in the Zebrafish Embryo. Nat. Commun..

[11] Sanchez Bosch P., Cho B., Axelrod J.D. (2024). Flamingo Participates in Multiple Models of Cell Competition. eLife.

[12] Petrova E., López-Gay J.M., Rhiner C., Moreno E. (2012). Flower-Deficient Mice Have Reduced Susceptibility to Skin Papilloma Formation. DMM Dis. Models Mech..

[13] Madan E., Pelham C.J., Nagane M., Parker T.M., Canas-Marques R., Fazio K., Shaik K., Yuan Y., Henriques V., Galzerano A. (2019). Flower Isoforms Promote Competitive Growth in Cancer. Nature.

[14] Li Y.Z., Gao L., Sun X.-L., Duan L., Jiang M., Wu Q.-F. (2025). Neural Cell Competition Sculpting Brain from Cradle to Grave. Natl. Sci. Rev..

[15] Merino M.M., Rhiner C., Lopez-Gay J.M., Buechel D., Hauert B., Moreno E. (2015). Elimination of Unfit Cells Maintains Tissue Health and Prolongs Lifespan. Cell.

[16] Marques-Reis M., Hauert B., Moreno E. (2024). Azot Is Not Essential for Loser Cell Elimination in a Time-Dependent Manner. J. Exp. Pathol..

[17] Ohsawa S., Sugimura K., Takino K., Igaki T. (2012). Imaging Cell Competition in Drosophila Imaginal Discs. Methods Enzymol..

[18] Gazzo D.V., Zartman J.J., Govin C.M. (2024). Calcium Imaging in Drosophila. Calcium Signaling.

[19] Rabinovich D., Mayseless O., Schuldiner O. (2015). Long Term Ex Vivo Culturing of Drosophila Brain as a Method to Live Image Pupal Brains: Insights into the Cellular Mechanisms of Neuronal Remodeling. Front. Cell. Neurosci..

[20] Segura R.C., Cabernard C. (2023). Live-Cell Imaging of Drosophila Melanogaster Third Instar Larval Brains. J. Vis. Exp..

[21] Huang C., Maxey J.R., Sinha S., Savall J., Gong Y., Schnitzer M.J. (2018). Long-Term Optical Brain Imaging in Live Adult Fruit Flies. Nat. Commun..

[22] Martin J.L., Sanders E.N., Moreno-Roman P., Jaramillo Koyama L.A., Balachandra S., Du X., O’Brien L.E. (2018). Long-Term Live Imaging of the Drosophila Adult Midgut Reveals Real-Time Dynamics of Division, Differentiation and Loss. eLife.

[23] Hubert A., Farkouh G., Harms F., Veilly C., Imperato S., Mercier M., Loriette V., Rouyer F., Fragola A. (2023). Enhanced Neuroimaging with a Calcium Sensor in Ex-Vivo Drosophila Melanogaster Brains Using Closed-Loop Adaptive Optics Light-Sheet Fluorescence Microscopy. J. Biomed. Opt..

[24] Tassara F.J., Barella M., Simó L., Folgueira Serrao M.M., Rodríguez-Caron M., Ispizua J.I., Ellisman M.H., de la Iglesia H.O., Ceriani M.F., Gargiulo J. (2025). Single Objective Light Sheet Microscopy Allows High-Resolution in Vivo Brain Imaging of Drosophila. bioRxiv.

[25] Sharpe J., Wong R.O. (2011). Imaging in Developmental Biology: A Laboratory Manual.

[26] Boto T., Tomchik S.M. (2024). Ex Vivo Brain Imaging in Drosophila. Cold Spring Harb. Protoc..

[27] Moreno E., Fernandez-Marrero Y., Meyer P., Rhiner C. (2015). Brain Regeneration in Drosophila Involves Comparison of Neuronal Fitness. Curr. Biol..

[28] Gutiérrez-García A., Marques-Reis M., Hauert B., Moreno E. (2025). Temporal Dynamics of Azot Expression Following Traumatic Brain Injury. bioRxiv.

[29] (2025). *Vienna Drosophila Food Recipe*; Vienna Biocenter. https://www.viennabiocenter.org/fileadmin/user_upload/VBCF/VDRC/VDRC_normal_fly_food_recipe_Sep2019.pdf.

[30] Wu J.S., Luo L. (2006). A Protocol for Dissecting Drosophila Melanogaster Brains for Live Imaging or Immunostaining. Nat. Protoc..

